# An analysis of interactions between three structurally diverse anthocyanidins, as well as their glucosides, and model biological membranes, albumin, and plasmid DNA

**DOI:** 10.1038/s41598-023-39470-2

**Published:** 2023-08-09

**Authors:** Anita Dudek, Paulina Strugała-Danak, Teresa Kral, Martin Hof, Hanna Pruchnik

**Affiliations:** 1 Department of Physics and Biophysics, Wrocław University of Environmental and Life Sciences, C. K. Norwida 25, 50-375 Wrocław, Poland; 2 Department of Biophysical Chemistry, J. Heyrovsky Institute of Physical Chemistry of the Czech Academy of Sciences, Dolejskova 3, 18223 Prague 8, Czech Republic

**Keywords:** Biophysics, Chemical biology, Plant sciences

## Abstract

The aim of the study is to investigate the differences in the interaction of three structurally diverse anthocyanidins, namely peonidin, petunidin, and delphinidin, as well as their glucosides with model biological membranes, human albumin, and plasmid DNA in order to look into their structure–activity relationships. Fluorimetric studies, as well as ATR-FTIR analyses, were jointly used in order to determine the changes observed in both the hydrophilic and hydrophobic layers of cell-mimic membranes (MM) which reflected the membrane lipid composition of tumour cells and red blood cell membranes (RBCM). Our results showed that anthocyanins and anthocyanidins can cause an increase in the packing order of the polar heads of lipids, as well as interact with their deeper layers by reducing the fluidity of lipid chains. The results presented here indicate that all compounds tested here possessed the ability to bind to human serum albumin (HSA) and the presence of a glucose molecule within the structures formed by anthocyanidin reduces their ability to bind to proteins. Using fluorescence correlation spectroscopy, it was demonstrated that the compounds tested here were capable of forming stable complexes with plasmid DNA and, particularly, strong DNA conformational changes were observed in the presence of petunidin and corresponding glucoside, as well as delphinidin. The results we obtained can be useful in comprehending the anthocyanins therapeutic action as molecular antioxidants and provide a valuable insight into their mechanism of action.

## Introduction

Anthocyanins are water-soluble plant pigments belonging to the flavonoid group. Due to their diverse structure and occurrence, they are well known to possess a long list of beneficial health properties. Anthocyanins demonstrate anti-cancer^1^, anti-diabetic^2^, anti-neurodegenerative^3^, anti-inflammatory effects^4^ and prevent cardiovascular diseases^5^. There are numerous studies which emphasize that the beneficial effects of anthocyanins are closely related to their antioxidant potential, including neutralizing free radicals, both reactive oxygen and nitrogen species, as well as their ability to chelate metals^6^. Similar to other compounds in the flavonoid group, antioxidant properties can be provided by the presence of different numbers of hydroxyl groups located in the structure of anthocyanins^7^. Apart from the hydroxyl substituent, the anthocyanin molecule has the potential to undergo glycosylation by attaching a sugar moiety such as glucose, fructose, mannose, or other similar sugars. The sugar substituent is mainly attached at the C3 position in the C ring or, occasionally, at the C5 or C7 position in the A ring. The structure of anthocyanidins, in addition to the previously mentioned hydroxylation and glycosylation processes^8^, can be also altered by the presence of acyl or methoxyl groups. All of these structural components have their impact on the molecular size, hydrophobicity, or bioavailability of anthocyanins. According to the literature, the activity and properties of anthocyanins may be significantly influenced by the structure of the molecule and the attached substituents. Moreover, the activity of different compounds may exhibit significant variations. For example, it was demonstrated that malvidin with additional sugar substituents showed higher antioxidant activity than malvidin. Moreover, similar differences were noted between the glucoside and galactoside of malvidin. The compound equipped with a sugar moiety in the form of glucose was a more effective antioxidant^9^. Other researchers observed different effects on lipid oxidation inhibition between compounds with different numbers of hydroxyl groups—cyanidin with two OH groups was a more effective antioxidant than delphinidin with three OH groups^10^. The diverse structure of the anthocyanin molecule also influences its ability to combine with other biomolecules such as a cell membrane, proteins or DNA. The ability to permanently and stably bind to these molecules can significantly alter transport, activity and bioavailability. The interaction of biologically active compounds with a cell membrane is a crucial step in presenting their properties. In the case of flavonoids, there are two potential ways of interacting with membranes. The first draws attention to absorption on the membrane surface and the occurrence of the interaction of hydrophilic parts of the compound with lipid heads through hydrogen bonds, and the second indicates contact of hydrophobic parts of both the compound and the non-polar parts forming the membrane core^11^. Although the exact mechanism of interaction between flavonoids as well as their derivatives and a biological membrane has not been completely explained, it has been shown that they can accumulate themselves in the hydrophobic part of the bilayer and reduce its fluidity^12^. Researchers focuses on how anthocyanins bind to DNA or RNA often explained this by the co-pigmentation process, i.e. the attachment of the aromatic ring of the compound to the nucleic acid complex through intercalation between two closely packed base pairs^13^. Moreover, the similarities in the structure of anthocyanins and clinically used intercalating compounds, such as actinomycin D and mitomycin C, are also highlighted^14^. Interestingly, studies showed that the formation of anthocyanin-DNA complexes can protect molecules from both oxidative stress and free radicals, thus avoiding oxidative damage to DNA and increasing the stability of anthocyanin^15^.

The main aim of our research was to verify whether structural differences in the structure of the 6 anthocyanidins peonidin (Pn), petunidin (Pt) and delphinidin (Dp) and their corresponding glucosidic forms: peonidin 3-*O*-glucoside (Pn 3-glc), petunidin 3-*O*-glucoside (Pt 3-glc) and delphinidin 3-*O*-glucoside (Dp 3-glc) affect their interaction with biomolecules such as a biological membrane, proteins and plasmid DNA. To investigate the impact of structure on the compound activity, we studied anthocyanins differing in the number and substitution site of hydroxyl groups in the B ring and the presence of a sugar group at the C3 position in the A ring were selected (Table 4). The fluorimetric studies with using probes allowed us to localize anthocyanidins in the membrane and to determine changes in the fluidity of both the hydrophobic and hydrophilic parts of a membrane. Moreover, using attenuated total reflectance Fourier transform infrared spectroscopy (ATR-FTIR), the structure and fluidity of the phospholipid layer was determined. A mimetic membrane (MM), which has a similar lipid composition to both a cancer cell and human erythrocyte membranes (RBCM), was chosen as a model for liposome membranes. An observation of natural fluorescence quenching in regard to human serum albumin (HSA), as one of the main blood transport proteins, made it possible to determine the affinity of these compounds for this protein and thus enabled a closer look at both pharmacokinetics and distribution of anthocyanins in blood. The fluorescence correlation spectroscopy technique was used to evaluate the interaction between anthocyanins and a single molecule of plasmid DNA. That is due to the fact that investigating the impact which anthocyanins have on the physical properties of liposome membranes and their interactions with HSA and plasmid DNA is essential.

## Results

### Interaction with the lipid membranes

#### Fluorimetric study

**Figure 1:**
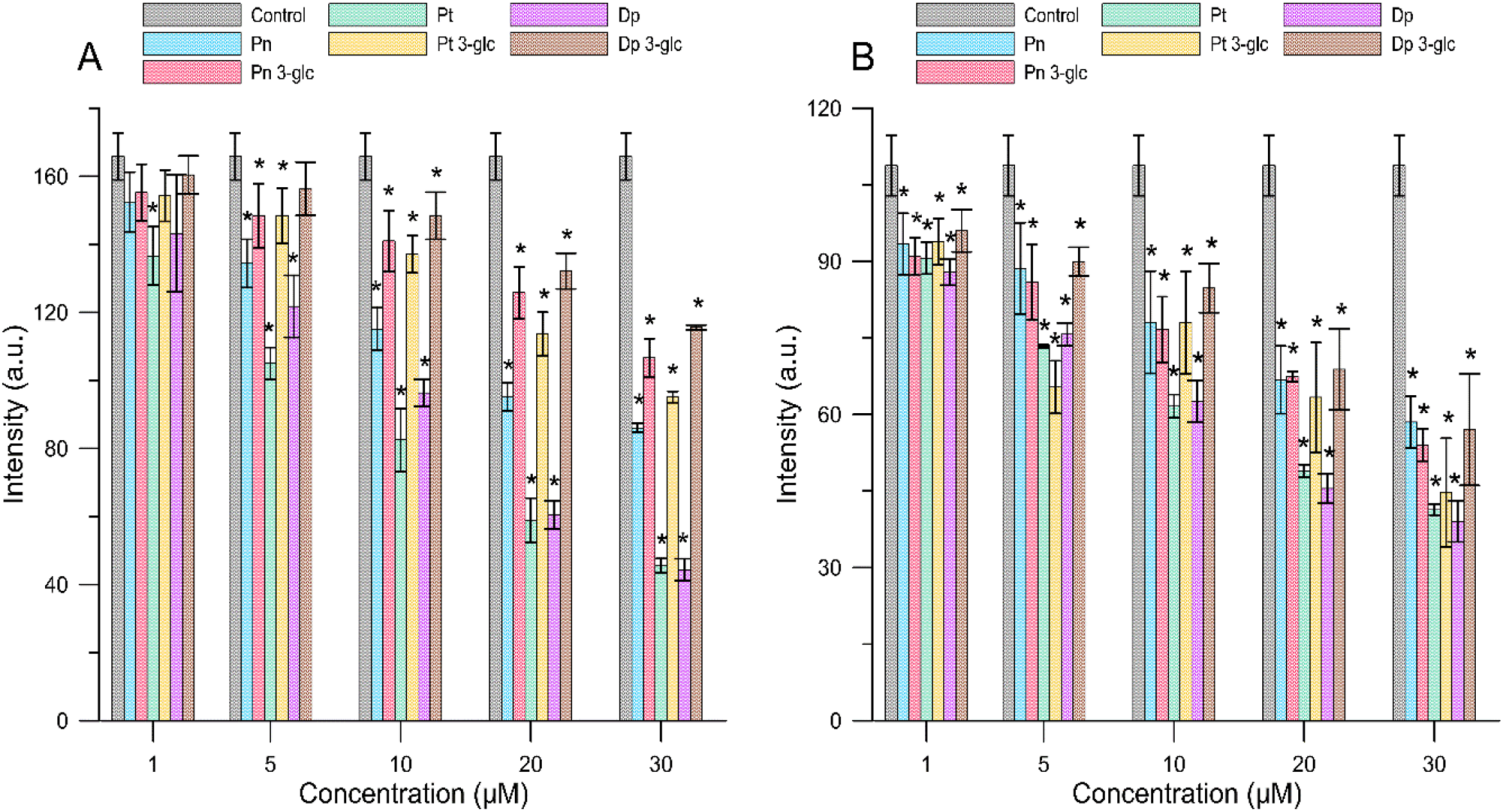
The fluorescence intensity values of the MC540 for MM (**A**) and RBCM (**B**) liposomes in the presence of different concentrations of anthocyanins The results are presented in the form of mean value ± standard deviation. Means labelled with asterisk (*) are significantly (p < 0.05) different from the control.

The fluorimetric study, involving fluorescence intensity and anisotropy measurements of MC540, TMA-DPH, and DPH probes, was conducted in order to determine the effect of anthocyanins differing in the number and location of hydroxyl groups and also sugar substituents on the properties of the hydrophilic and hydrophobic regions of both MM and RBCM liposomes. The changes in fluorescence intensity of the MC540 probe (Fig. 1) provided information about the ordering of lipids in the polar region of a membrane. A decrease in its fluorescence intensity relative to the control indicated that anthocyanins were able to incorporate into the hydrophilic part of the bilayer. The relationship focused on how anthocyanins decreased in the fluorescence intensity of the MC540 probe and thus induced an increase in the ordering of molecules in the outer layer of lipid membranes was concentration-dependent. The largest decrease in intensity for all tested anthocyanins was observed at a 30 µM concentration. Depending on their molecular structure, the anthocyanins differently affected structural changes in the polar region of the bilayer. In the case of MM, anthocyanins without a sugar substituent higher rigidized the hydrophilic region of a membrane compared to glycosides. The changes (expressed as percentage) in fluorescence intensity relative to the control (Supplementary Materials Table S1) for the most effective compounds and at the highest concentration used, namely delphinidin and petunidin, were 73.2 ± 5.3% and 72.5 ± 3.3%, respectively, whereas for their glucosides 30.3 ± 0.2% and 42.6 ± 0.7%. For RBCM liposomes, the greatest changes (expressed as percentage) in fluorescence intensity were observed for delphinidin and petunidin (at a concentration of 30 µM)—64.1 ± 6.6% and 61.9 ± 1.6%, respectively. Moreover, smaller differences between aglycones and glucosides were observed for RBCM liposomes than for MM liposomes. Those were 47.6 ± 9.1% for delphinidin glucoside and 58.9 ± 14.0% for petunidin glucoside.

**Figure 2:**
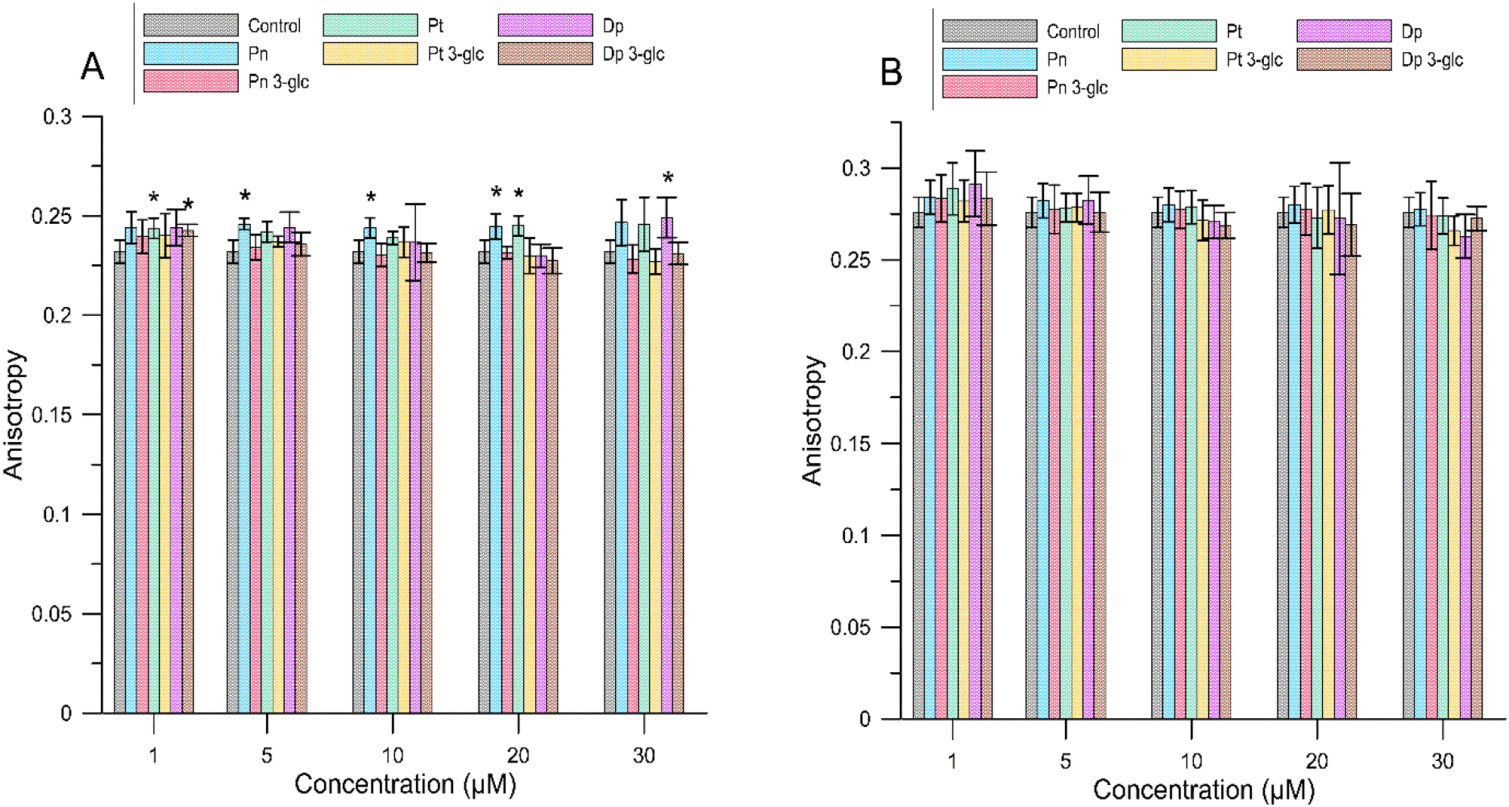
The anisotropy value for TMA-DPH for MM (**A**) and RBCM (**B**) liposomes in the presence of different concentrations of anthocyanins. The results are presented as mean value ± standard deviation. Means labelled with an asterisk (*) are significantly (p < 0.05) different from the control.

**Figure 3:**
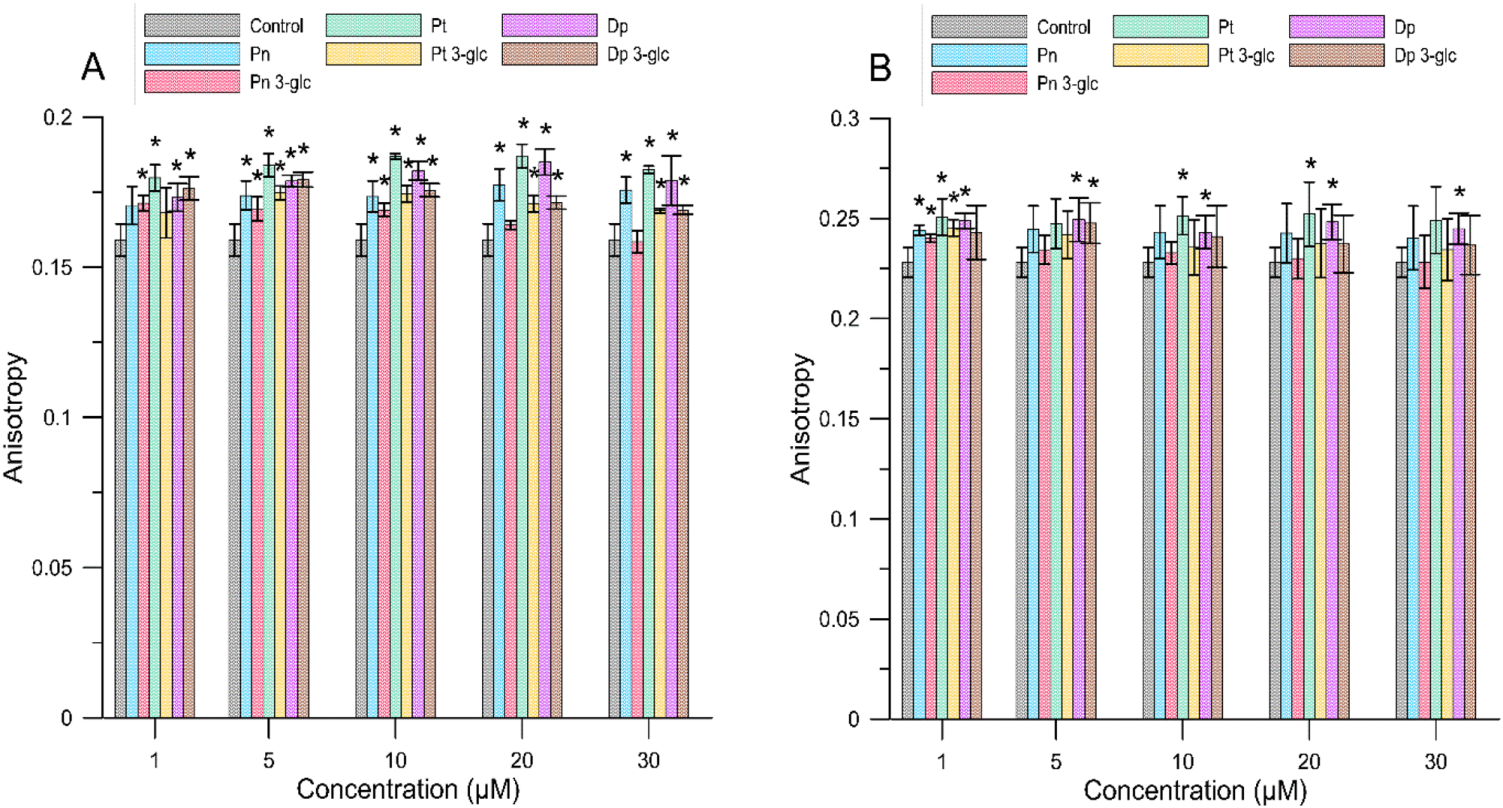
The anisotropy value for DPH for MM (**A**) and RBCM (**B**) liposomes in the presence of different concentrations of anthocyanins. The results are presented as mean value ± standard deviation. Means labelled with an asterisk (*) are significantly (p < 0.05) different from the control.

By determining the fluorescence anisotropy of the DPH and TMA-DPH probes, we were able to provide information about changes in the fluidity of lipid alkyl chains in the hydrophobic part of the bilayer where both probes were localized. Anisotropy values relative to controls for MM and RBCM liposomes are shown in Figs. 2 and 3. A statistical significant increase in anisotropy (p < 0.05) compared to the control sample was not observed for all compounds, indicating that not all anthocyanins reduced membrane fluidity and thus rigidity of the hydrophobic region of the lipid bilayer. Based on the graphs shown below (Figs. 2 and 3) and the table in the supplementary materials (Tables S2 and S3), statistically speaking, more significant increase in anisotropy was observed for the DPH probe than for TMA-DPH. It may suggest that the preferred anthocyanin site of incorporation/localization in a lipid membrane is located in its deeper, hydrophobic region, below the fourth carbon of the alkyl chains. The anthocyanins tested reduced the lipid fluidity of the bilayer formed from MM liposomes to a greater extent (by 17.6 ± 0.4% for the DPH probe, and TMA-DPH 7.4 ± 0.3%) than RBCM liposomes (by 10.5 ± 0.7% for the DPH probe, and TMA-DPH 4.7 ± 0.2%). Furthermore, no clear concentration-dependent relationship was observed. The erythrocyte membrane is structured differently from the mimetic membrane and, this might explain a weaker effect of anthocyanins on this type of a membrane^16^. The fluorescence anisotropy values for the TMA-DPH probe for all compounds were similar, and not statistically significant, with one except for Pt, Pn and Dp 3-glc and Dp at the specific concentrations, a fact which suggests that the structure of a molecule does not clearly affect the interaction of the compound with this inter-phase region of the membrane. For the DPH probe, the compound that mostly influenced the lipid rigidity in the hydrophobic region of a membrane was petunidin, for which the highest percentage of an increase in anisotropy relative to the control was 17.6 ± 0.4% (at a concentration of 20 µM) for MM liposome and 10.5 ± 0.7% at the same concentration for RBCM liposome.

The fluorescence quenching method of the DPH probe determined the partition coefficient expressed as *K*_*d*_ for the tested anthocyanins against both MM and RBCM. A high value of the dissociation constant for the hydrophobic part of the bilayer demonstrated low affinity of the compound for the liposome membrane. The results of this experiment are shown in Table 1. Our study showed that aglycones have higher affinity for liposome membranes than glycosides, although this relationship is more significant for MM than RBCM. The value of *K*_*d*_ decreased according to the sequence: Pt ≥ Dp > Pn > Pt 3-glc >> Dp 3-glc > Pn 3-glc for MM and Dp > Dp 3-glc > Pt ≥ Pn > Pn 3-glc > Pt 3-glc for RBCM. The presented values of the constants of dissociation *K*_*d*_ based on quenching of the hydrophobic DPH probe, together with the previously discussed changes in the anisotropy of the DPH probe, further confirmed that anthocyanins can affect the hydrophobic region of the membrane bilayer of the liposome.

**Table 1:** Values of dissociation constants *K*_*d*_ for the tested anthocyanins.

Compound	*K*_*d*_ for MM (µM)	*K*_*d*_ for RBCM (µM)
Pn	12.97 ± 2.92	17.54 ± 4.45
Pn 3-glc	114.28 ± 17.22	28.19 ± 5.11
Pt	10.43 ± 2.45	15.34 ± 4.01
Pt 3-glc	29.26 ± 5.43	46.94 ± 6.8
Dp	11.53 ± 2.07	9.89 ± 1.96
Dp 3-glc	93.57 ± 19.51	13.42 ± 3.05

Measurements were performed at 37 °C. The results are presented as mean value ± standard deviation.

### Attenuated total reflectance Fourier transform infrared spectroscopy (ATR-FTIR) studies

In addition, ATR-FTIR spectroscopy was conducted in order to analyze structural changes in the hydrophilic and hydrophobic regions of the lipid bilayer (MM and RBCM) in the presence of anthocyanins. Table 2 and Table S4 show the normal vibration values for selected groups of bands (methylene, ester, phosphate and choline) which are specific markers of changes in the lipid membranes studied here.

**Table 2:** Selected bands of ATR-FTIR spectra of RBCM and RBCM + compounds (30 µM).

*Vibration cm^−1^	RBCM	RBCM + Pn	RBCM + Pt	RBCM + Dp	RBCM + Pn 3-glc	RBCM + Pt 3-glc	RBCM + Dp 3-glc
ν_as_(N–C)	971.32	971.85	971.30	970.72	971.83	971.74	971.15
ν_s_(PO_2_^−^)	1084.47 1065.10	1066.86	1085.15 1066.04	1083.15 1062.59	1063.00	1062.40	1059.71
ν_as_(PO_2_^−^)	1230.09	1217.34	1226.92	1228.42 1215.23	1214.32	1229.2	1233.13 1209.44
ν(C=O)	1737.78	1739.16	1739.02	1738.13	1738.67	1739.17	1740.18
ν_s_(CH_2_)	2851.88	2851.95	2851.94	2851.94	2852.21	2852.36	2851.75
ν_as_(CH_2_)	2922.83	2922.70	2922.86	2923.26	2923.00	2923.25	2922.44

*Vibrations: ν, stretching; s, symmetric; as, antisymmetric.

The choline group is located in the outermost part of the bilayer and has a characteristic as follows: clearly visible vibrational band ν_as_(C–N^+^(CH_3_)) at 970 cm^−1^ for a dry lipid film. In the case of RBCM bilayers, 971.32 cm^−1^ was registered, while for MM it was 970.89 cm^−1^, which is probably due to differences in the lipid composition of membranes (degree of hydration). In the case of RBCM bilayers, the value of the wave number increased slightly for most of the tested compounds, one exception being made for delphinidin (Dp) and Dp 3-glc. After the addition of the compounds, significant shifts of the bands from the phosphate group were also observed, the vibrational band ν_as_ (PO_2_^−^) was extremely sensitive to the change in polarity of the environment and the possibility of interaction through hydrogen bonding. An increase in the hydration of the lipid bilayer causes the maximum of the ν_as_ (PO_2_^−^) vibration band to shift toward lower wave numbers, which is a consequence of increasing the number of lipid phosphate groups interacting with water molecules. Particularly large changes were seen for Pn, Dp and Pn 3-glc, indicating that the presence of these compounds increases the hydration of the MM bilayer and RBCM. Both Dp and Dp 3-glc compounds also caused significant shifts and separation of the phosphate group band into less and more hydrated subbands. Similar to the ν_as_ (PO_2_^−^) band, the ν(C=O) band also exhibited high sensitivity to changes in ambient polarity. The shift of the band toward higher values of wave numbers corresponded to the vibration of the C=O group characterized by weaker and/or fewer contacts with water molecules. This is the result produced by virtually all compounds studied, especially Dp 3-glc (Fig. S1). This may indicate a limited access of water molecules due to binding in the polar region of the bilayer. The last area analyzed was the region of hydrocarbon chains. The stretching vibration bands of the methylene group (ν(CH_2_)) had the highest intensity of all the bands of the phospholipid and lied at about 2920 and 2850 cm^−1^, for the vibration of ν_as_(CH_2_ ) and ν_s_ (CH_2_), respectively. It was found that in the case of a RBCM membrane, the stretching vibration bands of the methylene group (ν(CH_2_)) in the presence of all compounds were slightly shifted toward higher values of wave numbers, especially for Dp, Pn 3-glc, and Pt 3-glc. Interestingly, for Pd 3-glc, we observe an extremely slight decrease in vibrational frequency (Table 2). However, we did not observe a clear broadening of the bands, so we can assume that the number of *gauche* conformations along the C–C bonds of lipid alkyl chains did not increase (Fig. S1). Thus the fluidity in the hydrophobic region of the RBCM bilayer did not change significantly. Whereas slightly larger changes were seen for the MM membrane, for all compounds, there was a slight increase in the vibrational frequency (ν(CH_2_)) of the methylene group of the MM membrane lipids. In summary, IR studies, including fluorimetric studies, indicated that anthocyanins interact with the lipid bilayer, especially with its polar region. The degree of change depends on the structure of the compound tested here.

### Interaction with protein: fluorescence of HSA

**Figure 4:**
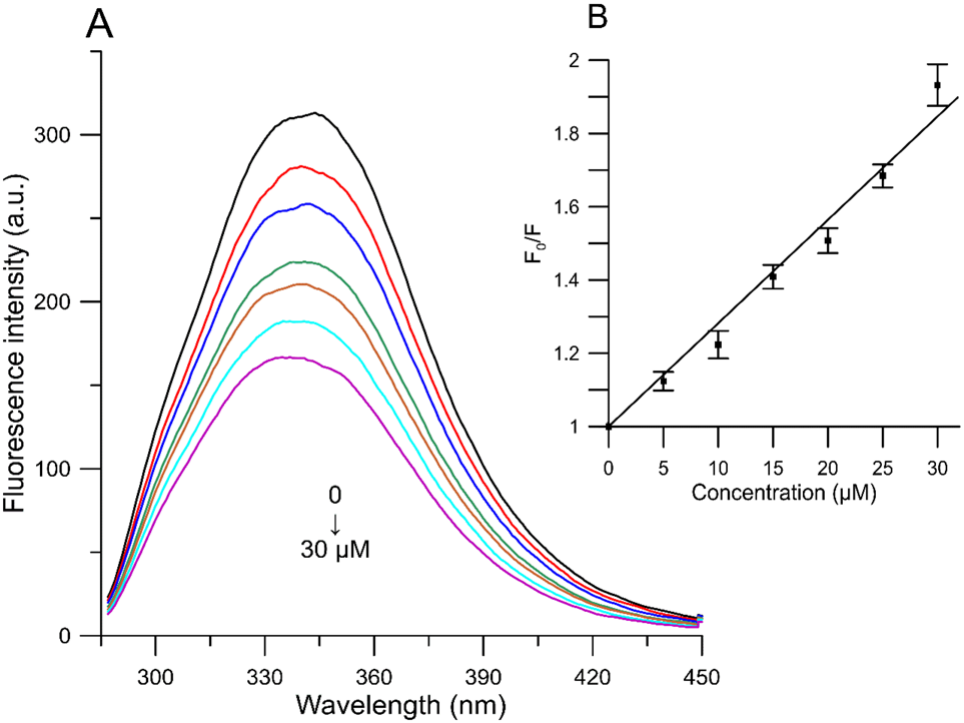
(**A**) Emission spectra of HSA in the presence of various concentrations of peonidin and (**B**) Stern–Volmer plots of *F*_*0*_*/F* against the concentration of study molecules (HSA = 1.5 × 10^–5^ M, λ_ex_ = 280 nm 37 °C).

To study the interaction of human serum albumin with anthocyanins, the phenomenon of quenching the natural fluorescence of albumin in the presence of anthocyanins was used. The appearance of the quenching effect (Fig. 4) indicated the possibility of binding anthocyanins to HSA. It was observed that the distribution of fluorescence depends on the concentration of anthocyanins. When the highest concentration of compounds was used, the fluorescence intensity was the lowest. The addition of anthocyanins (5–30 µM) resulted in a decrease in the fluorescence intensity, and the highest, namely 51% decrease relative to the control occurred in the presence of peonidin at a concentration of 30 µM (Fig. 4).

For a more detailed characteristic of the albumin-anthocyanin complexes during the fluorescence quenching process, the Stern–Volmer equation was applied. Based on the equations (Eqs. 4, 5), the Stern–Volmer quenching constant (*K*_*SV*_), the binding constant (*K*_*b*_), and the number of binding sites (*n*) were all determined (Table 3).

**Table 3:** Stern Volmer constant (*K*_*SV*_), binding constant (*K*_*b*_), and the number of binding sites (*n*).

Compound	*K*_*SV*_ × 10^3^ M^−1^	*K*_*b*_ × 10^3^ M^−1^	*n*
Pn	29.37 ± 1.09	96.33 ± 1.19	1.12 ± 0.04
Pn 3-glc	11.93 ± 1.61	9.37 ± 0.99	0.91 ± 0.09
Pt	25.99 ± 1.77	39.43 ± 4.20	1.03 ± 0.03
Pt 3-glc	5.50 ± 0.94	3.84 ± 0.41	0.92 ± 0.04
Dp	22.46 ± 1.00	30.68 ± 7.24	1.03 ± 0.05
Dp 3-glc	20.09 ± 2.57	11.30 ± 3.36	0.96 ± 0.03

Measurements were performed at 37 °C. The results are presented as mean value ± standard deviation.

According to the values summarized in Table 3, we observed significant differences in the ability to bind to albumin between the tested compounds. Aglycones were marked by better abilities to form complexes with albumin (higher values of binding constants) compared to glycosides. The presence of a sugar substituent significantly reduced the ability to bind to HSA, which is reflected in all three parameters. The highest affinity for binding to HSA among all anthocyanins was shown by peonidin, which is also confirmed by the emission spectrum (Fig. 4).

### Interaction of the anthocyanins with plasmid DNA

**Figure 5:**
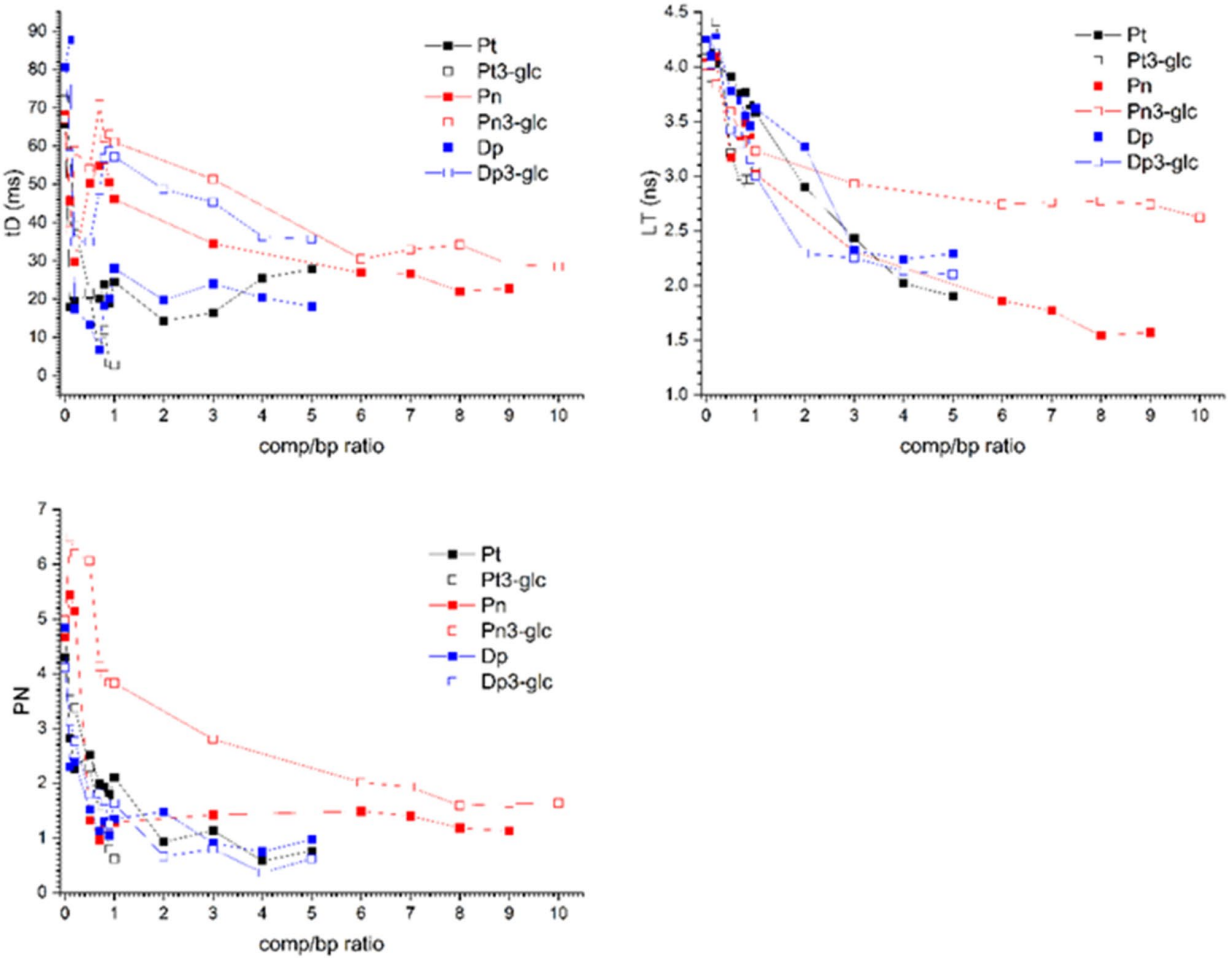
Diffusion time (tD), particle number (PN) and lifetime (LT) dependence on comp/ /bp ratio. The pHbApr-1-Neo plasmid (10 kbp and contour length 3.4 μm) was labelled with PicoGreen (CPico/CDNAbp = 0.02).

Studies focused on interaction between plasmid DNA molecules and anthocyanins were conducted using fluorescence correlation spectroscopy with the function of single photon counting, enabling tracking of dynamics changes at the level of single molecules, while tracking changes in fluorescence lifetime. The measurement results, using time-correlated single-photon counting fluorescence correlation spectroscopy TCSPC-FCS, which describe parameters such as Particle Number (PN, ± 0.1), lifetime (LT, ns, ± 0.1 ns) and diffusion time (tD, ms, ± 10% of its value) at each point) are presented in Fig. 5. The major, about a 20-fold decrease in the diffusion time (tD) (from about 70 ms to 3.5 ms), was observed for petunidin 3-*O*-glucoside (Pt 3-glc) at the concentration of comp/bp = 1. Simultaneously, a decrease in the PN value from 5 to about 0.7, and a decrease in the LT value from 4.25 ns to about 3.0 ns for this compound was observed. This is the only compound with a comp/bp range of 0 to 1. For the comp/bp = 0.8 ratios a tenfold decrease in the diffusion time (tD) (from about 70 ms to 7.0 ms) was observed for delphinidin followed by a decrease in the PN value from 5 to about 1.2 and a decrease in the LT value from 4.25 ns to about 3.5 ns. For comp/bp within the range of 0.8–1.0, an increase in the tD value was observed, resulting in only a twofold decrease compared to the initial plasmid diffusion time, reaching a plateau of 25–28 ms for the concentration ratio for values within the range of 1.0–5.0. Simultaneously, no changes in the PN value and a decrease in the LT value from 3.5 ns to about 2.3 ns were observed. The characteristics of changes in the tD, PN and LT values for petunidin are extremely similar to those observed for delphinidin. However, for petunidin a gradual but only 3.5-fold decrease in tD was finally observed. Simultaneously, within the tested range of comp/bp from 0 to 5, a continuous decrease in the values, both LT (from 4.25 to 2.3 ns) and PN (from 5.0 to about 0) was observed. In the case of peonidin, the obtained results indicated a threefold decrease in tD (from 70 ms to about 22 ms); thus, similarly to delphinidin and petunidin; however, for almost twofold higher comp/bp ratio equal to 9. Moreover, in the case of peonidin (Pn), a continuous and almost threefold, decrease in the LT value was observed (from 4.25 to 1.5 ns). Changes in the PN value have a two-stage character. In the range of ratios 0 < comp/bp < 1, a major fivefold decrease in PN was observed (from 5 to about 1). For concentration ratios in the range 5 < comp/bp < 9, the PN reached the plateau value of about 1.2. For the Dp 3-glc, the 2.5-fold decrease of the tD value was observed in the tested concentration range of the comp/bp (from 0 to 5). Changes in the LT value had a two-stage character. In the range of ratios 0 < comp/bp < 2, a major decrease in LT was observed (from 4.25 ns to about 2.25 ns). For concentration ratios within the range 2 < comp/bp < 5, the LT reached the plateau value of about 2.1 ns (within experimental error). Results obtained for the Pn 3-glc indicated the 2.5-fold decrease of the tD value observed in the tested concentration range of the comp/bp (from 0 to 10). Changes in the LT value had a two-stage character. In the range of ratios 0 < comp/bp < 1, a major decrease in LT was observed (from 4.25 ns to about 3.25 ns). Furthermore, along with an increase in the concentration ratios comp/bp, from 1 to 10, a minor further decrease of LT was observed up to the value of about 2.75 ns.

## Discussion

Our fluorimetric studies shown that anthocyanins can alter the properties of the hydrophilic region of the lipid bilayer by increasing the ordering of the polar regions of the lipids and also can incorporate into the deeper regions of the membrane by decreasing the fluidity of the hydrocarbon chains of lipids. Such structural modifications of membranes induced by the presence of anthocyanins can enhance their antioxidant properties, including reducing permeability and fluidity of membranes which may, therefore, lead to a reduction in the penetration of free radicals into the membrane and provide better protection against oxidation. Fluorimetric studies also provide information about the anthocyanins' favoured localization in membranes. In studies of flavonoids with different amounts of hydroxyl groups, it was found that they were mainly localized in the transition phase containing polar parts, and it was noted that the more polar luteolin and myrcetin localized closer to the surface of membranes, in the water/lipid interphase, and less polar compounds such as flavone and chrysin closer to the hydrophobic core of the membrane^17^. Similar connections were reached by other researchers^11^ who investigated the interactions between flavonoids and the DPPC membrane and found that flavonoids containing numerous hydroxyl groups bound more firmly to the membrane, which may be related to the potential for hydrogen bond formation. In our study, we observed a similar relationship only in the case of the mimetic membrane, where compounds with three and two OH groups—delphinidin and poeonidin, respectively—had the greatest effect on increasing the packing of molecules in the outer parts of the membrane. Such a relationship was not observed for the localization of compounds in the deeper parts of the membrane. The number and presence of hydroxyl groups as well as sugar substituents may affect both the relative hydrophobicity of a molecule, but also its planarity and spatial structure. In research Tsuchiya (2010) it was found that substitution of the hydroxyl group in the C-ring for a sugar substituent significantly reduced the ability of flavonoids to react with the lipid membrane, showing that its location in the C-ring may be essential for interaction with membranes^18^. Our study reveals greater affinity of aglycones than glycosides for both types of the membrane which may be related to the ability to form intramolecular and intermolecular hydrogen bonds between the hydroxyl group of anthocyanins and lipid molecules^19^. Other studies confirm that *O*-glycosylation of cyanidin significantly limited its ability to modify the hydrophobic region of the membrane, and the cyanidin aglycone displayed the highest affinity for the model POPC membrane^20^. It is suggested that the presence of sugar substituents in the structure of the molecule increases their size thereby making it difficult for the molecules to penetrate into deeper regions of the membranes^21^. On the other hand, glycosylated at position 7, the flavonoid derivatives narinegin and erdiocytol showed greater affinity for the liposome membrane than the corresponding, aglycones which might be explained by changes in the degree of torsion of the molecule due to glycosylation ^22^. In our previous studies it was observed that other anthocyanins, malividine and its sugar derivatives, can induce changes in the degree of packing of the polar parts of the lipids of the mimetic membrane, while they have no or very minor effect on the hydrophobic parts of the membrane^23^. Membrane rigidification and reduction of membrane permeability may be particularly relevant for cancer cells, whose membranes are generally more liquid than those of regular cells^24,25^. The greater fluidity of tumour cell membranes is explained by an increase in the degree of unsaturation of phospholipid acyl chains and a decrease in the compositional ratio of cholesterol to phospholipids^26^. In addition, anthocyanins inhibit lipid oxidation processes and possess antioxidant properties as confirmed in our previous work^27^ and several other studies^5,28–31^. If anthocyanins exhibit the ability to localize in the membrane and change its structural properties, as well as show the ability to protect cells from oxidation processes caused by free radicals, this could make them extremely valuable substances for protecting cells from an oxidative stress and thus the potential development of cancer cells. On the other hand, the evidence we presented on the interaction of anthocyanins with the model cell membrane of human erythrocytes, allows us to get a comprehensive picture of such interactions and the localization of anthocyanins in the membrane of authentic cells occurring in a human body. Moreover, it responds to the necessity of exploring this type of research and understanding the exact mechanisms of interaction between natural substances in the organism to take advantage of their therapeutic properties.

The ability of anthocyanins to bind to a blood transport protein such as human serum albumin (HSA) provides opportunities to understand both the pharmacokinetics of such a complex and potential future applications of anthocyanins as therapeutic substances. Moreover, this protein can protect the connected compound against degradation and loss of antioxidant properties^32^. Taking into account structural differences other than the sugar substituent of an individual compounds, it could be concluded that the presence of hydroxyl and methoxy groups could also have an impact on the ability to bind to proteins. In the case of aglycones, as the number of hydroxyl groups increased, the bonding and quenching constants decreased, but this did not greatly affect the number of binding sites. The presence of a glucose moiety in the anthocyanin structure decreased the parameters of binding to HSA which could be explained by a decrease in the hydrophobicity of the compound and may indicate the important role of hydrophobic interactions between anthocyanins and proteins^33^. The relationship between the number of hydroxyl groups and the affinity of anthocyanins for HSA was pointed out by Tang et al.^34^ and Cahana et al.^35^. It was indicated that the affinity rises with an increase in the number of OH groups and highlights the value of attractive electrostatic interactions, such as hydrogen bonding between polar groups. Other studies showed that the binding strength of anthocyanins, both aglycones, and glucosides, is not influenced by ring hydroxylation or demethylation of sugar residues^36^. However, the same study also found that delphinidin had the strongest affinity for HSA. The researchers emphasized that glycosylation affected the increased affinity for albumin in the 3-*O*-glucoside of peonidin and petunidin, but also significantly reduced the binding of delphinidin glucoside, which had a tenfold lower affinity for this protein compared to aglycone.

By following the changes in tD, PN and LT, we examined whether DNA molecules can make a complex with anthocyanins at a single molecule level and if it could lead to the conformational change of the entire plasmid molecule and, finally, if this could result in a formation of the tightly packed anthocyanin/DNA assembly (DNA folding). Changes in diffusion time of the polyamine molecule, which is plasmid DNA, correspond to its conformational changes. The increase or decrease in tD is the result of changes in the hydrodynamic radius of the plasmid. PN is a number that refers to the number of fluorescent events within the confocal volume (CV). In the case of the presented data, the DNA molecules concentration was 1 nM. The PN parameter for this concentration should be 0.7 ± 0.1. For the presented plasmid, the initial overestimated values of the PN were equal 5. The PN parameter reaches values close to 0.7 when the entire DNA molecule changes its conformation to a more packed, “point-like” molecule. Thus, changes in the PN value are an indicator of changes between the relaxed (unfolded) and compacted (folded) DNA plasmid conformation, as previously reported^37^ (Fig. 5). Fluorescence lifetime (FL) refers to the average time that a chromophore spends in an excited state. If there are processes that affect the direct environment of the chromophores, there are usually reflected in the lifetime changes. All the compounds tested here interacted with plasmid DNA. Evidence for this comes from the changes in diffusion time (tD), particle number (PN) and lifetime (LT). Petunidin 3-*O*-glucoside (Pt 3-glc) interacts with plasmid molecules in the comp/bp range from 0 to 1. Changes in tD, LT and PN prove strong DNA packing, which, in turn, proves its strong interaction with Pt3-glc molecules. Compared to the other anthocyanins presented here, Pt3-glc is the most potent compound. For the comp/bp concentration range up to 0.8, changes in plasmid tD in the presence of Delphinidine (Dp) and Petunidine (Pt) are about 2- and 3.5-fold lower compared to those obtained for Pt 3-glc. This indicates conformational changes of the plasmid that do not lead to so tight packing of the plasmid in this concentration range, both for Dp and Pt. Additionally, in the case of Dp; for comp/bp concentrations > 0.8, an increase in tD was observed with a continuous decrease in LT. This can be attributed to the destabilization of the DNA helix at high concentrations of Dp and what had already been reported^38^ The obtained tD, PN values for peonidine (Pn) indicate conformational changes of the plasmid conformation but do not result in its folding, even for comp/bp values almost twofold higher than in the case of Dp and Pt. At the same time, a major decrease in the LT value was obtained, which indicates a strong interaction of Pn with two binding sites of the PicoGreen (PG) molecule which simultaneously binds as an intercalator and minor groove binder^39,40^. In the case of Delphinidin 3-*O*-glucoside (Dp 3-glc), the obtained results indicated even more subtle conformational changes in the entire plasmid molecule. For the Dp3-glc, the determined tD changes were more than 5 times smaller than those caused by interactions with the most active Pt3-glc. The compound whose interaction resulted in the smallest conformational changes of the DNA molecule was peonidine 3-*O*-glucoside (Pn 3-glc). This was confirmed both by changes in the tD and, most importantly, by changes in PN. The PN values obtained for the comp/bp range (0 to 10) did not correspond to the point-like molecule assumption, i.e. they did not approach values close to 0.7. At the same time, the characteristics of the decrease in the LT value indicated an unambiguous interaction with the PicoGreen binding sites.

## Conclusion

In our comprehensive study, we have demonstrated that various structural characteristics of anthocyanins play a significant role in their interactions with other biomolecules, including protein, plasmid DNA, and lipids. The fluorimetric analysis revealed that anthocyanins have the capacity to modify the physicochemical properties of both hydrophilic and hydrophobic regions of model lipid membranes. This was also confirmed in a way of conducting ATR-FTIR analysis. The determined dissociation constant (*K*_*d*_) for anthocyanins with the liposome membrane confirmed the interaction of anthocyanins with the hydrophobic region of this lipid membrane and indicated its lower permeability and fluidity. Both anthocyanidins and glucosides forms demonstrated their ability to bind to human serum albumin, and the presence of a sugar substituent in a molecule limited this ability. The complexes formed by anthocyanin-protein can determine both pharmacokinetics and distribution of these compounds which were formed by anthocyanins in a body. The results presented here give a clear answer to the question whether a plasmid DNA molecule can form a stable complex with the tested anthocyanins. Pt 3-glc, Dp and Pt compounds strongly interact with DNA, forming highly packed assemblies. The influence of the anthocyanins studied here on the conformation of the DNA molecule, running from its tight packing to a slightly changed conformation, can be presented in the following order: Pt 3-glc > Dp > Pt > Pn > Dp 3-glc > Pn 3-glc.

Our research has provided an insight into how anthocyanins interact with these biomolecules which are crucial for both availability and distribution of biologically active compounds in a body. Demonstrating the ability possessed by selected anthocyanins to bind to biological membranes, major blood transporters of proteins such as human albumin and plasmid DNA, as well as having a deeper insight into the structure–activity relationships, both contribute to increases in the significance of anthocyanins as potential therapeutics and may be the first step in understanding their biological activity.

## Materials and methods

### Materials

Anthocyanins: peonidin, petunidin, delphinidin, and their glucosides peonidin 3-*O*-glucoside , petunidin 3-*O*-glucoside , and delphinidin 3-*O*-glucoside (Table 4)—Extrasynthese (Lyon Nord, France). Lipids: 1-Palmitoyl-2-oleoylphosphatidylcholine (POPC), 1-palmitoyl-2-oleoylphosphatidylethanolamine (POPE), and 1-stearoyl-2- oleoylphosphatidylserine (SOPS)-Avanti Polar Lipids (Delfzijl, The Netherlands). Cholesterol—Sigma-Aldrich (St Louis, MO, USA). Probes: 1,6-diphenyl-1,3,5-hexatriene (DPH), Merocyanine 540 (MC540), *N*,*N*,*N*-trimethyl-4-(6-phenyl-1,3,5-hexatrien-1-yl)phenylammonium *p*-toluenesulfonate (TMA-DPH)—Molecular Probes (Eugene, OR, USA).

**Table 4:** The chemical structure of the compounds studied.

Compound	Abbreviations	R_1_	R_2_	R_3_
Peonidin	Pn	–OH	–OCH_3_	–H
Peonidin 3-*O*-glucoside	Pn 3-glc	Glc	–OCH_3_	–H
Petunidin	Pt	–OH	–OH	–OCH_3_
Petunidin 3-*O*-glucoside	Pt 3-glc	Glc	–OH	–OCH_3_
Delphinidin	Dp	–OH	–OH	–OH
Delphinidin 3-*O*-glucoside	Dp 3-glc	Glc	–OH	–OH

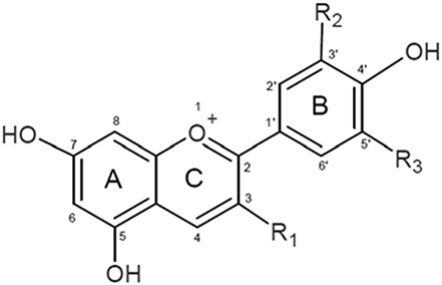

### Liposome preparation

Two types of liposomes were used to study interactions with biological membranes: liposomes mimicking the composition of cancer cell membranes (mimic membrane liposome—MM) and liposomes formed from lipids extracted from human blood erythrocyte membranes (red blood cell membrane liposome—RBCM). MM liposomes were formed from four types of lipids: POPC—48%, POPE-24%, SOPS—8%, and cholesterol—20% and dissolved in chloroform, based on our previous work^41^. The RBCM liposome was according to the methodology described previously^42^ with minor modifications^43^. Both lipids, dissolved in chloroform (MM liposome) and n-decane (RBCM liposome), were evaporated under a nitrogen atmosphere, and then dried in a desiccator for 90 min under a vacuum. The obtained dry lipid film was hydrated in phosphate buffer, pH 7.4, obtaining a concentration of 0.1 mg/ml, and then vortexed for one minute until a homogeneous, milky suspension was observed. The mixture was sonicated (Sonic, Milano, Italia) for 15 min keeping the sample in a bath of ice water, thus providing a cooling temperature of 0 °C. The suspension was then processed (extrusion) through polycarbonate filters with pore diameters of 100 and 200 nm using a Lipex extruder (Evonik). Liposomes, which had been prepared according to this procedure, was used in fluorimetric studies.

### Interaction with the lipid membranes

#### Fluorimetric studies: MC540, TMA-DPH and DPH probes

Fluorimetric studies were applied to determine the packing order of lipids in the MM and RBCM liposomes and examined the effect of anthocyanins on the physical properties of the hydrophilic and hydrophobic region of liposome membranes. To investigate these properties three fluorescent probes were used: MC540, TMA-DPH, and DPH. MC540 localizes to the outer, hydrophilic part of the membrane, within the region of the glycerol skeleton^44^. Changes in the fluorescence intensity of this probe were formulated as the relative change in intensity to the control as a function of anthocyanins concentration. The TMA-DPH and DPH probes are supposed to locate nearer to the interface and in the hydrophobic region, respectively^45^. The measurement of changes in the anisotropy of the TMA-DPH and DPH probes allowed us to reflect the mobility of the fluorescent probes in the membrane, and therefore to determine the fluidity of the bilayer^46^. Fluorescence anisotropy was calculated using the formula^47^:

1 $$A = \frac{{I_{\|} - GI_{ \bot } }}{{I_{\|} + 2GI_{ \bot } }}$$

where ${I}_{\| }$ and $I_{ \bot }$ are the intensity of fluorescence in parallel and perpendicular directions, to the plane of polarization of the excitation wave, respectively. *G* is an apparatus constant that depends on the emission wavelength.

The phenomenon of quenching the fluorescence of the DPH probe by biologically active substances enabled us to evaluate the partition coefficient (*K*_*d*_, dissociation constant) of anthocyanins with both MM and RBCM liposome membranes. For this purpose, changes in the fluorescence intensity of this probe were checked and the coefficient was calculated using the equation^48^:

2 $$\frac{1}{\left(\frac{{F}_{0}}{F}\right)-1} =\frac{{K}_{d}}{[ liposomes]}\times \frac{1}{[anthocyanins]}+\frac{1}{\left[liposomes\right]}$$

where F_0_—fluorescence intensity in the absence of anthocyanins (control sample), *F*—fluorescence intensity in the presence of anthocyanins, *[liposomes]*—concentration of liposomes in mg/ml, *[anthocyanins]*—concentration of anthocyanins tested mg/ml. The linear dependence obtained from the graph of *1/[(F*_*0*_*/F)—1]* versus *1/[anthocyanins]* was used to determine the slope coefficient (slope divided by an intercept) of the straight line and further to determine the *K*_*d*_ value according to the Tammela and others^30^.

Liposomes (the concentration of lipids in the sample was 0.1 mg/ml) were incubated for 30 min with the appropriate probe (dissolved in DMF, 1 µM concentration), and then the tested anthocyanins were added (final concentration of 1 to 30 µM). The control sample consisted of liposomes and a fluorescent probe. Fluorescence was measured on a fluorimeter (Cary Eclipse, Varian, San Diego, USA) at 37 °C. The excitation wavelengths λ_ex_ and emission wavelengths λem respectively: MC540—λ_exc_ = 540 nm, λ_em_ = 590 nm; DPH—λ_exc_ = 360 nm, λ_em_ = 425 nm. nm; TMA-DPH—λ_exc_ = 340 nm, λ_em_ = 430 nm.

#### ATR- FTIR studies

In addition, Fourier attenuated total reflectance infrared spectroscopy was used to study the effect of anthocyanins on the fluidity of MM and RBCM bilayers. Samples for the study were prepared according to the method described in an earlier paper^49^. The test compounds were dissolved in ethanol and added to a lipid solution (MM or RBCM), the mixture was applied to a ZnSe crystal and the solvent was carefully removed under vacuum. The lipid content in each sample was 0.1 mg, and the concentration of the compound was 30 μM. Measurements of the dry and hydrated film were performed at 37 °C using a Nicolet 6700 FT-IR spectrometer (Thermo Fisher Scientific, Waltham, MA). Analysis of the obtained spectra was performed using OMNIC software (Thermo Nicolet).

### Interaction with biomolecules

#### Fluorescence quenching of albumin

The phenomenon of quenching the natural fluorescence of human serum albumin (HSA) was used to investigate the potential interaction of anthocyanins with this protein. Analysis of the interaction of studied compounds with HSA was performed according to the work of Strugała et al. ^50^ Anthocyanins with concentrations ranging from 1 to 30 µM were added to HSA dissolved in phosphate buffer (1.5 × 10^–5^ M) and then incubated at 37 °C for 20 min. Emission spectra were performed on a fluorimeter Cary Eclipse (Varian, San Diego, USA) at 37 °C. HSA fluorescence was excited with a wavelength of λ = 280 nm, the emission was measured in the range from 286 to 460 nm, and the fluorescence intensity was read at 345 nm where the emission maximum of HSA was located. To obtain the appropriate fluorescence intensity values, emission data was corrected by inner filter effect according: to Eq. (3) as suggested by Lakowicz^47^.

3 $${F}_{corr}={F}_{obs}\cdot {10 }^{(Aex+Aem)/2}$$

where F_corr_ and F_obs_ are the corrected and observed fluorescence intensities of HSA, respectively; A_ex_ is the absorbance value at the excitation wavelength and A_em_ is the absorbance value at the emission wavelength.

The Stern–Volmer equation was used to thoroughly describe the quenching mechanism of natural fluorescence and to determine the bindings parameters of HSA: anthocyanin complexes:

4 $$\frac{{F}_{0}}{F}=1+{K}_{q}{\tau }_{0}\left[Q\right]=1+{K}_{SV}[Q]$$

where *F*_*0*_—fluorescence intensity in the absence of anthocyanins (control sample), F—fluorescence intensity in the presence of anthocyanins, *K*_*SV*_—Stern–Volmer quenching constant (*K*_*SV*_ = *K*_*q*_·τ_0_), [Q]—concentration of quencher (tested compound), *K*_*q*_—bimolecular quenching constant, *τ*_*0*_—a lifetime of the fluorophore in the absence of quencher the fluorescence lifetime of a biopolymer is calculated as 5 × 10^–9^ s^51^.

The apparent binding constants (*K*_*b*_) and the number of binding sites (*n*) can be calculated using the following equation:

5 $$\mathit{log}\left[{(F}_{0 }-F\right)/F]=\mathit{log}{K}_{b }+n\mathit{log}[Q]$$

F_0_ and F are the fluorescence intensities before and after the addition of the quencher. From the linear plot of *log (F*_*0*_* − F)/F* versus *log [Q]*, the value of *K*_*b*_ was obtained from the intercept.

#### Interaction with DNA (time-correlated single-photon counting fluorescence correlation spectroscopy (TCSPC-FCS) measurements)

To evaluate the biological interaction with DNA molecules the anthocyanins were initially dissolved in ETOH (1 mM). That was necessary due to anthocyanins’ low aqueous solubility. Mixing of such solutions with the aqueous DNA solutions used in the studies never exceeded 1% concentration ETOH (v/v) in the final solution. The DNA plasmid was labelled by PicoGreen (CPicoGreen/CDNAbp = 0.02)^52^. The pHbApr-1-Neo plasmid (10 kbp and contour length 3.4 µm) was a generous gift from the laboratory of Prof. Maciej Ugorski (Department of Biochemistry and Molecular Biology, Wrocław University of Environmental and Life Sciences Wroclaw, Poland). PicoGreen-labelled DNA samples mixed with the appropriate amount of anthocyanins were left overnight and then measured. The increasing concentration of anthocyanins in the tested samples was expressed as the ratio of the molar concentration of anthocyanins per molar concentration of DNA base pairs in a given sample (comp/bp). The 100 µl samples were measured in Ibidi 8-well chambers. Each point on the graph is a single sample (average of 10 measurements). All measurements were performed at room temperature (25 °C). Time-correlated single-photon counting fluorescence correlation spectroscopy (TCSPC-FCS) was used to study the interaction of plasmid DNA at the single molecule level with all tested anthocyanins. Measurements were performed on Microtime 200 inverted confocal microscope (PicoQuant, Germany) with the pulsed diode laser (LDH-P-C-470 nm PicoQuant) providing 80 ps pulses at 40 mHz repetition rate, dichroic mirror 490DRLP and bandpass filter 515/50 (Omega Optical), and water immersion objective (1.2 NA, 60x) (Olympus). Low power of 1uW (at the back aperture of the objective) was chosen to minimize photobleaching and saturation. In the detection plane, a pinhole (50um in diameter) was used and the signal was collected by a single photon avalanche diode (SPAD, Microphoton Devices, Bolzano, Italy). Photon arrival times were stored using fast electronics (Picoharp 300, PicoQuant) in time-tagged time-resolved recording mode. Two independent times were assigned to each detected photon: (1) a time after the beginning of the measurement and (2) a time after the previous laser pulse. The FCS data analysis was done using home-built routines (DevC++, Bloodshed Software and OriginPro70, OriginLab Corporation). The TCSPC data analysis was performed using SymPhoTime software (PicoQuant). Further details of the data evaluation are given elsewhere^53^.

### Statistical analysis

Data are shown as mean values ± standard deviation (SD). Statistical analysis was performed using the program Statistica 12.0 (StatSoft, Kraków, Poland). The results were analyzed by one-way ANOVA followed by Duncan test, *p* values < 0.05 were considered statistically significant.

### Supplementary Information

Supplementary Information.

## Data Availability

The datasets used and/or analyzed during the current study available from the corresponding author on reason able request.
