# Supplementary material for: An analysis of interactions between three structurally diverse anthocyanidins, as well as their glucosides, and model biological membranes, albumin, and plasmid DNA
Source: Sci Rep. 2023 Aug 9;13:12883. doi: 10.1038/s41598-023-39470-2 (PMC10412636; doi:10.1038/s41598-023-39470-2)

**An analysis of interactions between three structurally diverse anthocyanidins, as well as their glucosides, and model biological membranes, albumin, and plasmid DNA**

Anita Dudek^1^, Paulina Strugała-Danak^1,*^,Teresa Kral^1,2^,Martin Hof^2^, Hanna Pruchnik^1^

^1^ Department of Physics and Biophysics, Wrocław University of Environmental and Life Sciences, C. K. Norwida 25, 50-375 Wrocław, Poland

^2^ Department of Biophysical Chemistry, J. Heyrovsky Institute of Physical Chemistry of the Czech Academy of Sciences, Dolejskova 3, 18223 Prague 8, Czech Republic

**SUPPLEMENTARY MATERIALS**

**Table S1.** Summary of percentage changes in the fluorescence intensity of the MC540 probe relative to the control sample for ML and RBCL. Positive values indicate a decrease in fluorescence intensity compared to the control sample.

| Compound | Concentration (µM) | Changes relative to the control (%) | |
| --- | --- | --- | --- |
|  |  | **MM** | **RBCM** |
| Pn | 1  5  10  20  30 | 8.06±0.46  18.97±1.00  30.57±1.64  42.61±1.87  48.10±0.74 | 14.16±0.91  18.51±1.87  28.21±3.62  38.60±3.83  46.20±3.99 |
| Pn 3-glc | 1  5  10  20  30 | 6.40±0.34  10.52±0.67  14.98±0.94  24.13±1.45  35.68±1.88 | 16.40±0.66  20.98±1.80  29.53±2.47  38.00±0.57  50.34±2.93 |
| Pt | 1  5  10  20  30 | 17.63±1.11  36.62±1.63  50.22±5.62  64.46±7.12  72.51±3.32 | 16.68±0.58  32.60±0.15  43.35±1.60  55.02±1.41  61.93±1.58 |
| Pt 3-glc | 1  5  10  20  30 | 6.95±0.35  10.52±0.57  17.35±0.69  31.49±1.78  42.63±0.74 | 13.68±0.65  39.91±3.15  28.28±3.62  41.75±7.08  58.91±14.03 |
| Dp | 1  5  10  20  30 | 13.66±1.64  26.57±1.99  41.91±1.76  63.50±4.28  73.23±5.31 | 19.22±0.56  30.39±0.90  42.45±2.77  58.15±3.73  64.08±6.62 |
| Dp 3-glc | 1  5  10  20  30 | 3.27±0.12  5.73±0.30  10.51±0.55  20.30±0.93  30.30±0.19 | 11.73±0.51  17.29±0.54  22.06±1.25  36.70±4.24  47.56±9.12 |

**Table S2.** Summary of percentage changes in the anisotropy of the TMA-DPH probe relative to the control sample for ML and RBCML. Negative values indicate an increase in anisotropy relative to the control, while positive values indicate a decrease in anisotropy relative to the control sample.

| Compound | Concentration (µM) | Changes relative to the control (%) | |
| --- | --- | --- | --- |
|  |  | **MM** | **RBCM** |
| Pn | 1 | -5.60±0.17 | -3.0±0.1 |
|  | 5 | -5.99±0.08 | -2.31±0.08 |
|  | 10 | -5.13±0.11 | -1.54±0.05 |
|  | 20 | -5.50±0.14 | -1.53±0.05 |
|  | 30 | -6.35±0.30 | -0.62±0.02 |
| Pn 3-glc | 1 | -3.26±0.11 | -2.75±0.12 |
|  | 5 | -0.96±0.03 | -0.59±0.03 |
|  | 10 | 0.69±0.02 | -0.56±0.02 |
|  | 20 | 0.19±0.003 | -0.61±0.03 |
|  | 30 | 1.63±0.05 | 0.65±0.04 |
| Pt | 1 | -5.05±0.11 | -4.68±0.23 |
|  | 5 | -4.34±0.09 | -0.95±0.03 |
|  | 10 | -2.97±0.04 | -1.05±0.03 |
|  | 20 | -5.53±0.11 | 1.13±0.07 |
|  | 30 | -5.92±0.33 | 0.70±0.02 |
| Pt 3-glc | 1 | -3.47±0.16 | -2.24±0.09 |
|  | 5 | -2.18±0.02 | -0.99±0.03 |
|  | 10 | -2.04±0.07 | 1.63±0.07 |
|  | 20 | 0.91±0.03 | -0.47±0.02 |
|  | 30 | -2.20±0.06 | 3.60±0.11 |
| Dp | 1 | -5.27±0.20 | -5.61±0.35 |
|  | 5 | -5.27±0.16 | -2.41±0.11 |
|  | 10 | -2.04±0.17 | 1.85±0.06 |
|  | 20 | 0.86±0.02 | 1.35±0.15 |
|  | 30 | -7.43±0.30 | 4.73±0.21 |
| Dp 3-glc | 1 | -4.62±0.05 | -2.73±0.14 |
|  | 5 | -1.60±0.04 | -0.01±0.00 |
|  | 10 | 0.26±0.005 | 2.57±0.07 |
|  | 20 | 1.99±0.06 | 2.52±0.16 |
|  | 30 | 0.41±0.01 | 1.15±0.03 |

**Table S3.** Summary of percentage changes in DPH probe anisotropy relative to the control sample for ML and RBCM. Negative values indicate an increase in anisotropy relative to the control sample.

| **Compound** | **Concentration (µM)** | **Changes relative to the control (%)** | |
| --- | --- | --- | --- |
|  |  | **MM** | **RBCM** |
| **Pn** | 1  5  10  20  30 | -7.22±0.27  -9.29±0.26  -9.08±0.27  -11.57±0.35  -10.44±0.25 | -6.97±0.08  -7.25±0.35  -6.59±0.36  -6.36±0.39  -5.30±0.35 |
| **Pn 3-glc** | 1  5  10  20  30 | -7.68±0.11  -6.50±0.15  -6.27±0.08  -3.12±0.03  -0.39±0.01 | -5.23±0.04  -2.72±0.08  -2.02±0.05  -0.79±0.03  -0.03±0.002 |
| **Pt** | 1  5  10  20  30 | -13.00±0.31  -15.66±0.33  -17.48±0.09  -17.59±0.36  -14.74±0.10 | -9.91±0.36  -8.46±0.42  -10.18±0.39  -10.51±0.67  -9.21±0.62 |
| **Pt 3-glc** | 1  5  10  20  30 | -5.67±0.28  -9.84±0.13  -9.71±0.15  -7.55±0.12  -6.11±0.03 | -7.54±0.13  -7.10±0.35  -7.11±0.42  -7.30±0.53  -7.24±0.48 |
| **Dp** | 1  5  10  20  30 | -8.87±0.24  -12.33±0.13  -14.45±0.24  -16.33±0.38  -12.45±0.57 | -9.06±0.14  -9.32±0.41  -6.55±0.22  -8.84±0.31  -7.33±0.24 |
| **Dp 3-glc** | 1  5  10  20  30 | -10.78±0.24  -12.62±0.18  -10.40±0.13  -7.80±0.10  -6.27±0.05 | -6.55±0.37  -6.14±0.25  -6.03±0.39  -6.20±0.38  -6.30±0.39 |

**Table S4.** Selected bands of ATR-FTIR spectra of MM and MM+compounds (30 μM).

| ***vibration**  **cm^-1^** | **MM** | **MM+Pn** | **MM+Pt** | **MM+Dp** | **MM+**  **Pn 3-glc** | **MM+**  **Pt 3-glc** | **MM+**  **Dp 3-glc** |
| --- | --- | --- | --- | --- | --- | --- | --- |
| **ν_as_ (N-C)** | 970.89 | 970.49 | 970.48 | 971.37 | 971.71 | 972.11 | 969.93 |
| **ν_s_(PO_2_^-^)** | 1087.47 | 1066.74 | 1066.39 | 1065.04 | 1065.04 | 1065.70 | 1079.59  1065.25  1045.76 |
| **ν_as_(PO_2_^-^)** | 1232.58 | 1215.07 | 1234.12 | 1200.43 | 1212.81 | 1233.81 | 1235.46  1207.36 |
| **ν(C=O)** | 1738.38 | 1739.67 | 1738.82 | 1738.10 | 1738.92 | 1739.35 | 1740.24 |
| **ν_s_(CH_2_)** | 2851.27 | 2851.89 | 2851.53 | 2851.65 | 2851.55 | 2851.58 | 2851.59 |
| **ν_as_(CH_2_)** | 2920.45 | 2921.46 | 2921.56 | 2921.33 | 2921.17 | 2921.94 | 2921.82 |

* vibrations: ν—stretching; s—symmetric; as—antisymmetric.

**Figure S1.** FTIR spectra of RBCM bilayers and of MM modified with of peonidin, petunidin, and delphinidin, and their glucosides concentration of compounds 30 µM; (A) C–H stretching regions of RBCM, (B) C–H stretching regions of MM, (C) carbonyl band of RBCM , (D) carbonyl band of RBCM.


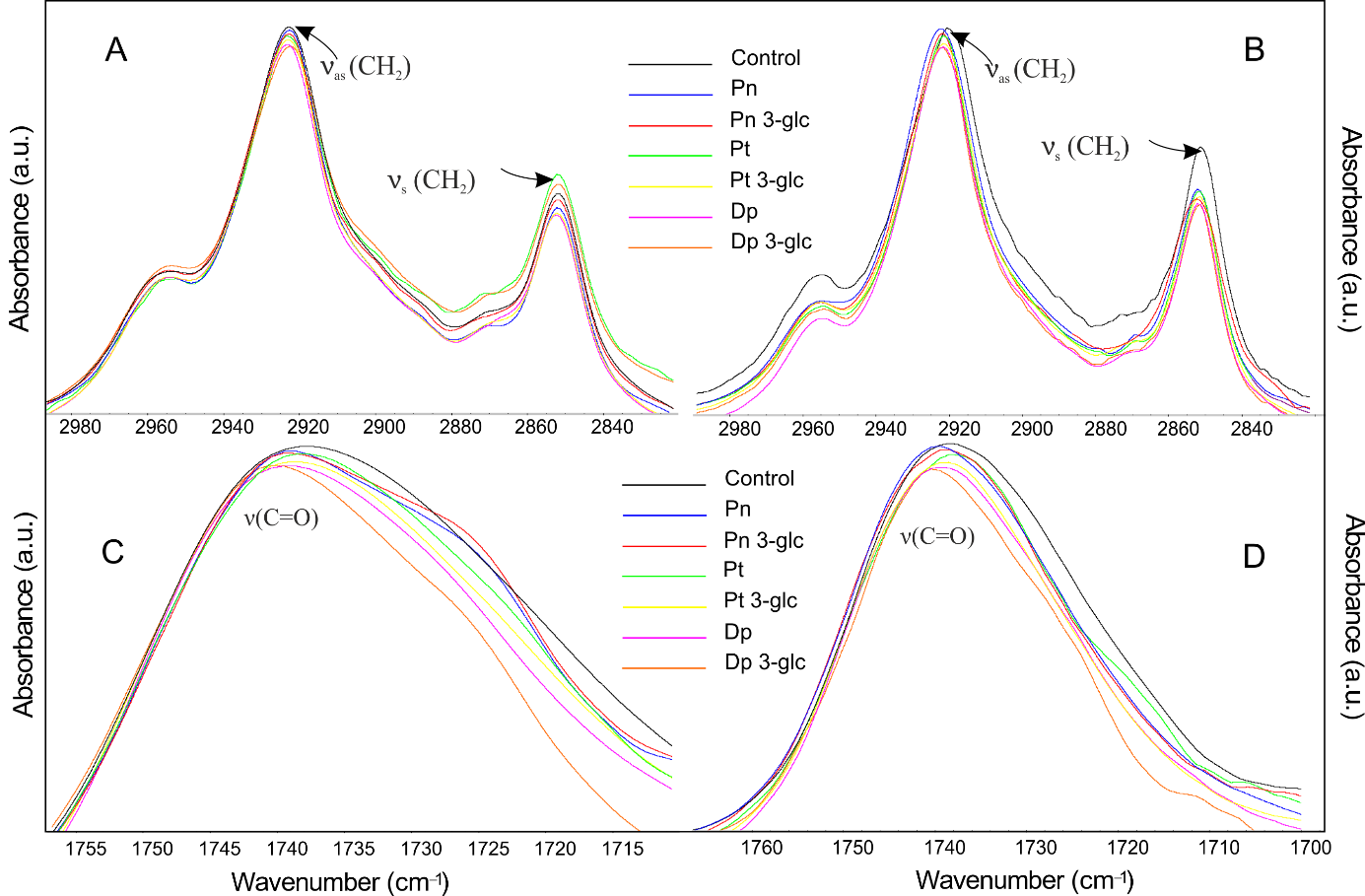

Supplement: Supplementary file 1 — Supplementary Information. [file 41598_2023_39470_MOESM1_ESM.docx]
